# UV radiation is the primary factor driving the variation in leaf phenolics across Chinese grasslands

**DOI:** 10.1002/ece3.862

**Published:** 2013-10-29

**Authors:** Litong Chen, Kechang Niu, Yi Wu, Yan Geng, Zhaorong Mi, Dan FB Flynn, Jin-Sheng He

**Affiliations:** 1 Key Laboratory of Adaptation and Evolution of Plateau Biota, Northwest Institute of Plateau Biology, Chinese Academy of Sciences23 Xinning Rd, Xining, 810008, China; 2 Department of Ecology, College of Urban and Environmental Sciences, and Key Laboratory for Earth Surface Processes of the Ministry of Education, Peking UniversityBeijing, 100871, China; 3 Institute of Evolutionary Biology and Environmental Studies, University of ZurichZürich, 8057, Switzerland

**Keywords:** Inner Mongolia, leaf functional traits, leaf phenolics, Tibetan Plateau, ultraviolet absorbing compounds, UV radiation

## Abstract

Due to the role leaf phenolics in defending against ultraviolet B (UVB) under previously controlled conditions, we hypothesize that ultraviolet radiation (UVR) could be a primary factor driving the variation in leaf phenolics in plants over a large geographic scale. We measured leaf total phenolics, ultraviolet-absorbing compounds (UVAC), and corresponding leaf N, P, and specific leaf area (SLA) in 151 common species. These species were from 84 sites across the Tibetan Plateau and Inner Mongolian grasslands of China with contrasting UVR (354 vs. 161 mW/cm^2^ on average). Overall, leaf phenolics and UVAC were all significantly higher on the Tibetan Plateau than in the Inner Mongolian grasslands, independent of phylogenetic relationships between species. Regression analyses showed that the variation in leaf phenolics was strongly affected by climatic factors, particularly UVR, and soil attributes across all sites. Structural equation modeling (SEM) identified the primary role of UVR in determining leaf phenolic concentrations, after accounting for colinearities with altitude, climatic, and edaphic factors. In addition, phenolics correlated positively with UVAC and SLA, and negatively with leaf N and N: P. These relationships were steeper in the lower-elevation Inner Mongolian than on the Tibetan Plateau grasslands. Our data support that the variation in leaf phenolics is controlled mainly by UV radiation, implying high leaf phenolics facilitates the adaptation of plants to strong irradiation via its UV-screening and/or antioxidation functions, particularly on the Tibetan Plateau. Importantly, our results also suggest that leaf phenolics may influence on vegetation attributes and indirectly affect ecosystem processes by covarying with leaf functional traits.

## Introduction

As a broad class of carbon-based secondary metabolites, leaf phenolics have received much attention due to their functional significances in plant ecological adaptation and evolution (Fraenkel 1959; Stafford 1991; Rozema et al. 1997; Jansen et al. 1998; Cockell and Knowland 1999; Agrawal and Fishbein 2008). Owing to their important roles in protecting plants from UV radiation, the functions of leaf phenolics have gained new interest (Agati and Tattini 2010; Pollastri and Tattini 2011). Ultraviolet-B (UVB) radiation reaching the Earth's surface increased significantly in the last several decades. And future variation in UV radiation resulting from rapid changes in global climate (e.g., cloud cover and aerosol) and land use may have more important consequences on terrestrial ecosystems than that caused by ozone depletion (Ballare et al. 2011). Therefore, leaf phenolics are likely to be key in the response of ecosystems to ongoing and future climate changes.

Phenolic compounds, which occur in nearly all plant species with concentrations up to 5–40% dry weight in leaves (Peñuelas et al. 2011), usually are assumed as a result of antiherbivore and/or antioxidants to UV radiation (UVR) (Coley et al. 1985; Rozema et al. 1997; Close and McArthur 2002; Agati and Tattini 2010). Yet, with ubiquitous occurrence of numerous kinds of phenolic compounds across diverse species and in heterogeneous environments (Levin 1971; de Jong 1995), it remains a challenge to clarify the functional roles of leaf phenolics in plant responses to environmental changes and adaptations to the long-term environmental stress, such as UVR.

To determine the potential drivers affecting the abundance and distribution of leaf phenolics, previous studies have focused on the effects of experimental manipulation of temperature (Kuokkanen et al. 2001; Pennycooke et al. 2005; Zvereva and Kozlov 2006; Albert et al. 2009), nutrient availability (de la Rosa et al. 2001; Sundqvist et al. 2012), and water availability (Turtola et al. 2005). In particular, many studies have consistently documented that excess light and UVB radiation have a crucial impact on the concentrations of plant phenolic compounds under controlled experiments (Hofmann et al. 2000; de la Rosa et al. 2001; Searles et al. 2002; Turtola et al. 2005; Dunn and Robinson 2006; Martz et al. 2007; Thines et al. 2007). In addition, plant phenolics have been found to function primarily in protecting leaves from photo-oxidative damage, rather than herbivore damage, by acting as antioxidants (Close and McArthur 2002; Ryan et al. 2002; Agati et al. 2009, 2011, 2012; Agati and Tattini 2010). However, the few studies measuring of leaf total phenolics in response to UVR in the variable natural environments, particularly at the large geographic and taxonomic scales, have found mixed results. In particular, long-term manipulations of UVR in the field have found only limited responses of leaf phenolics (Rozema et al. 2006), and field surveys have found that temperature, rather than UVR, may be the primary driver of leaf phenolics (Albert et al. 2009). Consequently, the functional roles of leaf phenolics in plant self-protection still remain to be identified with substantial data at the large geographic and taxonomic scales.

Grasslands cover about 40% of China's land surface (Kang et al. 2007), including the high-altitude Tibetan Plateau with a mean elevation of ≥4000 m and relatively low-altitude Inner Mongolian Plateau. Chinese grasslands provide an excellent opportunity to evaluate the response of leaf phenolics to UVR at the large geographic scale. We predicted that UV radiation could be the primary factor driving the variation in leaf phenolics across broad geographic range of Chinese grasslands. As a result, we further anticipated that plants from the Tibetan Plateau will have higher leaf phenolics than those in the Inner Mongolian Plateau due to strong UVB radiation at high altitudes. Therefore, we firstly test this hypothesis by relating leaf phenolics to field UVR and other climate factors across the broad geographic and taxonomic scales. We then explore the correlations between leaf phenolics and nutrient availability in the soil, leaf nutrient concentrations, and the growth trait specific leaf area (SLA). The relationships between leaf phenolics and these functional traits suggest that there is a fundamental trade-offs between defense, growth, and reproduction (Bazzaz and Grace 1997) from individual to population and species and even site level (Ackerly et al. 2000; Suding et al. 2003; Violle et al. 2007), yet such work has only been conducted at local scales or in glasshouse settings.

In this study, we investigated the variation in leaf total phenolics, UV-absorbing compounds (UVAC), corresponded SLA and leaf N and P in 342 populations belonging to 151 species. These species were from 84 sites across the Tibetan Plateau and Inner Mongolian grasslands with contrasting UVR (354 vs. 161 mW/cm^2^ on average). The study is the first to quantify the variation in leaf phenolics and relationships between leaf phenolics, environmental factors, and functional traits across a large group of species at the broad geographic scale. Specifically, we address the following questions: (i) Are leaf phenolics from Tibetan Plateau higher than those in Inner Mongolia? (ii) Is UVR the primary driver of leaf phenolic concentrations in comparison with other climatic factors and soil attributes at site level across the large geographic scale? (iii) How do leaf phenolics covary with leaf UVAC and other leaf functional traits such as SLA, leaf N, P, and N: P in contrasting flora regions at site and species levels?

## Material and Methods

### Site selection and species sampling

**Figure 1:**
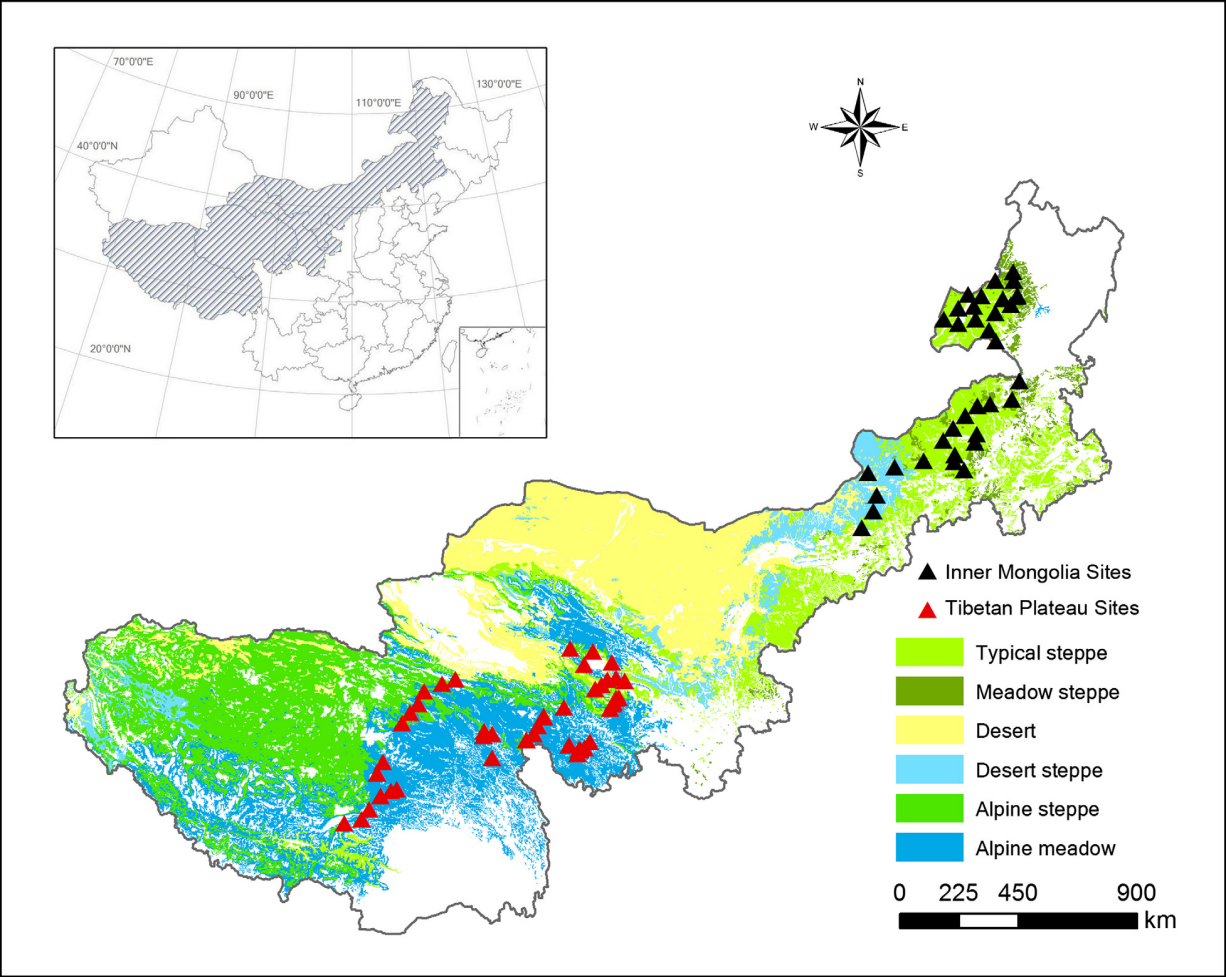
Vegetation map of the 84 sampling sites on the Tibetan Plateau (red triangles) and Inner Mongolia (black triangles) grasslands, selected from the Vegetation Map of China (Chinese Academy of Sciences 2001).

Based on our previous studies, we selected 84 sites in the grasslands of Tibetan Plateau and Inner Mongolia from late July to early August in 2006 and 2007 (Fig. 1, Table S1). These sites were selected to be representative of low-disturbance grazing in each area. Of the 84 sites, 28 were alpine meadow, 19 alpine steppes, 24 typical steppes, 7 desert steppes, and 6 meadow steppes (Table S1). The Tibetan Plateau grassland is characterized by *Kobresia*-dominated alpine meadow and *Stipa*-dominated alpine steppe. In contrast, the Inner Mongolian grasslands include *Stipa*-dominated typical steppe and desert steppe, and *Stipa*- and *Leymus*-dominated meadow steppe. At each site, sun-exposed and newly mature leaves (leaf blades for grasses) of five to ten plants of each species were sampled to determine SLA and concentrations of leaf C, N, P, leaf phenolics, and UVAC. Defining the occurrence of a species at a site as a population, we sampled 342 populations belong to 151 dominant species from 77 genera and 27 families (Fig. S1, Table S2). We recorded the geographic coordinates, elevation, climate data, and vegetable type for each site (Table S1). Descriptions of sampling protocol and measurements of leaf C, N, and P have been detailed in our previous studies (He et al. 2006a,b, 2008).

### Leaf total phenolics and UV-absorbing compounds measurements

Using 10 mg of ground dry leaf samples, we measured leaf total phenolics with the improved Folin–Ciocalteau method (Box 1983) and determined the concentrations of methanol-extractable ultraviolet-absorbing compounds (UVAC) following the procedure of Huttunen et al. (2005), respectively. Because UVAC such as flavonoids have either an intense absorption band around 260 nm with a weaker band above 300 nm (isoflavones, flavanones), or two bands of about equal intensity, one around 260 nm and the other around 340 nm (flavones) or 360 nm (flavonols) (Cerovic et al. 2002), the absorbing curve of the extracts was analyzed with the spectrophotometer (Pye Unicam UV4-100, Thermo Electron Corp., Waltham, MA, USA) between 240 and 360 nm. For simplicity, we use total integrated UV absorption between 240 and 360 nm to indicate UVAC of each individual sample.

### UV radiation measurements

We measured solar ultraviolet radiation (UV radiation) at each site on a cloudless day during the sampling campaign. We measured the daily UV radiation five times at each site (between 12 a.m. and 14 p.m. in the Inner Mongolian and Tibetan Plateau grasslands, respectively, Beijing Time) by an ultraviolet intensity meter (UVB dual-channel, Beijing Normal University Optical and Electronic Instrument Factory) with fixed wavelength at 290 nm. At large geographic scales, these measurements will be representative of the UV radiation at sampling sites. Due to the very extensive spatial scale of this work, it would have been impractical to install fixed UV-detecting spectroradiometers at each site.

### Soil attributes and climatic variables collections

At each site, three soil pits were excavated to collect soil samples. Soil samples for chemical analysis were air-dried, sieved (2 mm mesh), and handpicked to remove fine roots, and the remaining soil was ground using a ball mill (NM200; Restch, Haan, Germany). We measured soil inorganic carbon using an inorganic carbon analyzer (Calcimeter 08.53; Eijkelkamp, Giesbeek, Netherland), and soil total carbon (STC) using an elemental analyzer (VARIO ELIII; Elementar, Hanau, Germany), respectively. Soil organic carbon (SOC) was then determined as the difference between STC and SIC. Soil total nitrogen (STN) was also measured with an elemental analyzer (PE 2400 II CHN elemental analyzer; Perkin-Elmer, Boston, MA, USA). Soil sampling procedures and measurements have also been detailed in a previous study (Shi et al. 2012).

In situ measurements of temperature would have many advantages; however, it would be an enormous challenge to conduct such measurements of temperature because of the large number of sampling sites (84), and the broad distribution of these sites across Chinese grasslands, mainly in quite remote areas, where permanent climate stations solely for the purpose of this study are not practical. Therefore, the climate data used in this study including growing season temperature (GST, from May to September) and growing season precipitation (GSP) were compiled from the 1950–2000 temperature/precipitation records of a global climate database (Hijmans et al. 2005). Although the uncertainties in the climate database (Hijmans et al. 2005), it is currently the only available database at the broad geographic scale in Chinese grasslands. Greater availability of high-resolution temperature data at the biome scale would increase the accuracy of future work in this area.

### Statistical analyses

#### Calculations of the means of variables for site and species level

We calculated total leaf phenolics, UVAC, SLA, leaf N, P, and N: P ratio for each species by averaging individual plant measurements within species at each site to produce a species-by-site dataset. We then calculated these variables for site level by averaging the dominant species of each site to produce a dataset of site means. Finally, we calculated these variables at the species level by averaging measurements within species to produce a dataset of species means. We used log_10_ transformations to normalize the distributions.

#### Phylogenetic generalized least-squares (PGLS) regressions

To account for the phylogenetic independence between species (Freckleton et al. 2002), we used phylogenetic generalized least-squares (PGLS) regressions. The PGLS regressions can be used to compare multiple traits as predictors of leaf phenolics, as well as to calculate trait means in after controlling for phylogenetic relatedness and to analyze each bivariate combination of traits. These analyses were designed to determine whether region-level differences were being driven by higher order-taxonomic interactions. That is, whether responses of species are more heavily affected by taxon than the region in which they were sampled (Wright et al. 2002), following Wright et al. (2010). Using the method, we also tested for relationships among traits to account for phylogenetic associations following Holdaway et al. (2011). We used a Phylomatic phylogeny (Webb and Donoghue 2005) (Fig. S1) based on the order and family-level classifications of the Angiosperm Phylogeny Group. Branch lengths for this composite phylogeny were estimated according to the method of Grafen (1989) using the *ape* package in R (Paradis et al. 2004). We calculated the phylogenetic correlation (λ) using maximum likelihood (Freckleton et al. 2002). Lambda values range between 0 and 1, and traits that are phylogenetically independent have λ close to 0, whereas those that covary in proportion to their level of relatedness have λ close to 1. As PGLS parameters depend upon the order of the model, we modeled all traits combinations in both directions, to be conservative, only report those that are significant in both cases. Multiple and single regression in PGLS were carried out using the caper package in R.

#### Comparisons of leaf phenolics and UVAC between regions

We determined the differences of leaf phenolics and UVAC between regions using independent-sample *t*-tests at the site level and the PGLS at the species level. We employed one-way ANOVA with a Duncan post hoc test to test the differences of these traits among functional groups, vegetative types, and the nine most common genera at species level (Table S3), and the differences of leaf phenolics and UVAC among/within the same genus between Inner Mongolian and Tibetan Plateau grasslands (Fig. 4).

#### Determination of the primary driver of leaf phenolics

**Figure 2:**
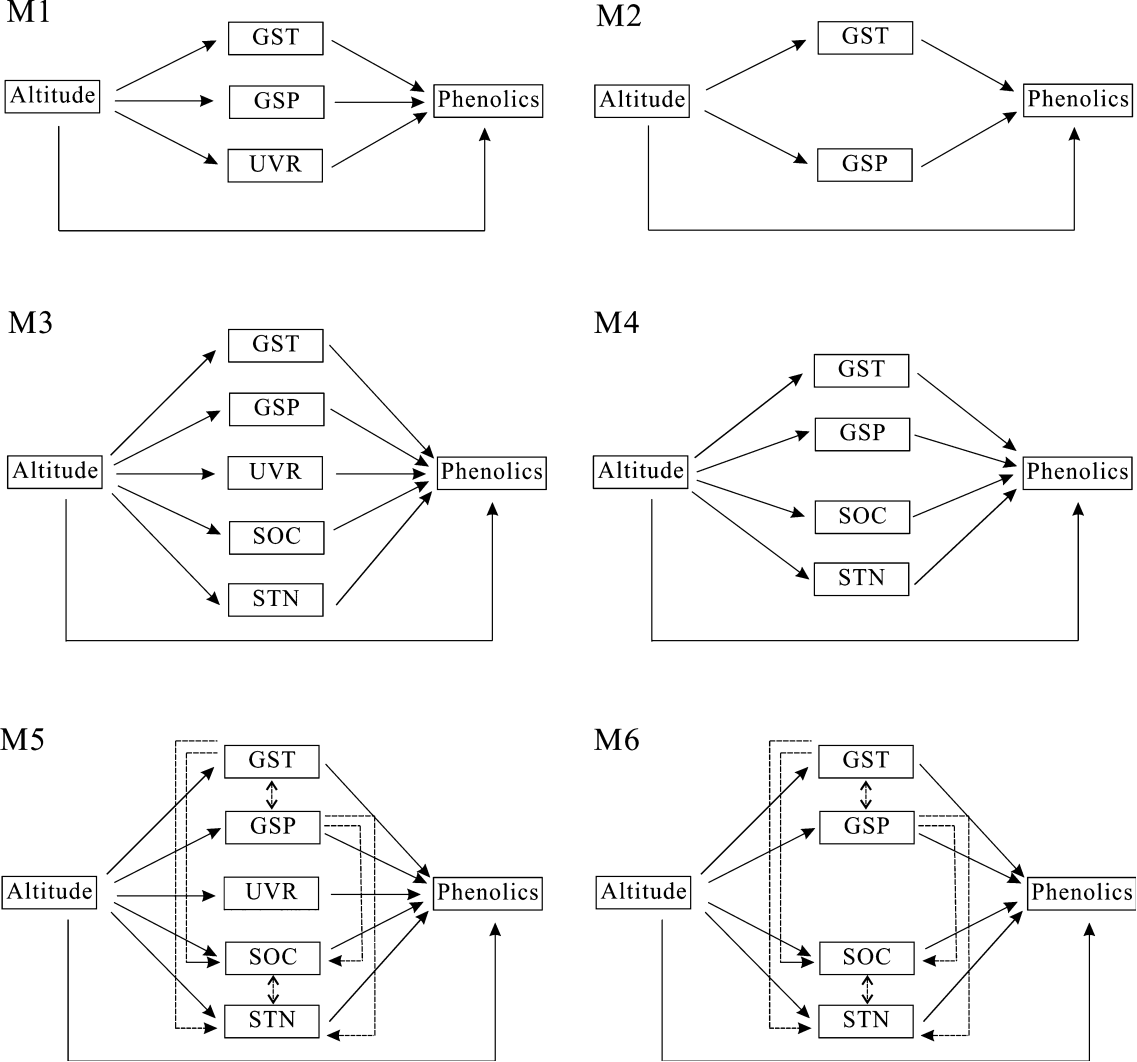
Structural equation models tested for combinations of growing season temperature and precipitation (GST, GSP), UV radiation (UVR), soil organic carbon (SOC), and soil total nitrogen (STN), in combination with altitude as predictors of environmental effects on leaf phenolics. Models were constructed to represent the effect of GST, GSP, UVR, SOC, or STN on leaf phenolics as functions of altitude, because the indices used here are largely affected by altitude. These models were chosen to test the degree to which environmental factors affect leaf phenolics, taking into account the correlations between these metrics.

We used linear regressions to understand the effects of climatic factors and soil attributes on leaf phenolics. Determining how these variables affect leaf phenolics is challenging because variables measured in the field are highly intercorrelated. We therefore performed structural equation modeling (SEM) with the site-level dataset using the *sem* package in R (Fox 2006) to further describe the interactive effects of environmental factors on leaf phenolics. Structural equation modeling analysis allowed us to construct alternative hypotheses for the relationships between climatic, soil, and other site variables, for the relationships between these variables and leaf phenolics. Our goal was to isolate the influence of UV radiation on leaf phenolic concentrations, after accounting for all other variables. After reconsidering the logic among environmental variables, we constructed six SEM models to focus only on the relationships of interest, in particular by comparing models with and without UVR (Fig. 2). Data were *z*-transformed before analysis, and alternative models were evaluated based on remaining unexplained error as expressed by root mean squared error (RMSE) and the SEM comparative fit index (CFI).

#### Determination of the relationships between leaf phenolics and other leaf traits

We explored the relationships between leaf phenolics and leaf traits (SLA, UVAC, leaf N, P, and N:P ratio) using linear regressions at site level, and the PGLS regressions at species level, respectively.

## Results

### Leaf phenolics from Tibetan Plateau were higher than those in Inner Mongolia

**Figure 3:**
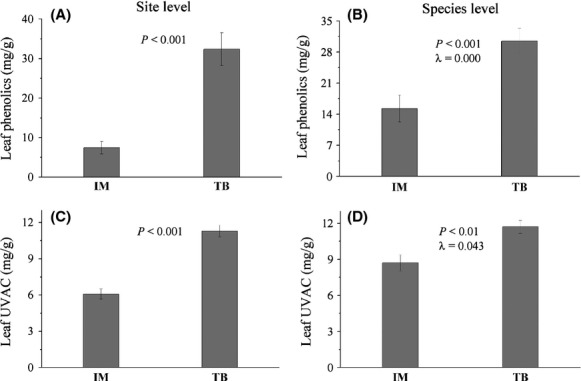
Leaf phenolics and UVAC for grassland plants sampled in Inner Mongolia (IM, 37 sites and 59 species) and Tibetan Plateau (TB, 47 sites and 101 species) at both site and species levels. The values indicate means ± SE, and means are compared using independent-sample *t*-test and PGLS regression at site and species level, respectively; lambda (λ) values indicate phylogenetic independence of traits.

Across the 84 field sites, leaf phenolics ranged from 0.61 to 190.55 mg/g with an average of 21.38 mg/g, and UVAC ranged from 1.65 to 16.39 mg/g with an average of 9.00 mg/g. In the Inner Mongolian grasslands, leaf phenolics and UVAC ranged between 0.61–35.80 and 1.65–13.52 mg/g with an average of 7.44 and 6.08 mg/g, respectively. By contrast, leaf phenolics and UVAC varied between 8.50–190.55 and 3.63–16.39 mg/g with an average of 32.36 and 11.30 mg/g on the Tibetan Plateau grasslands, respectively. At the site level, leaf phenolics and UVAC showed significant differences between Inner Mongolian and Tibetan Plateau grasslands. For example, the mean of leaf phenolics on the Tibetan Plateau grasslands was over four time higher than those in the Inner Mongolian grasslands (32.36 vs. 7.44 mg/g) (Fig. 3A), and the mean of leaf UVAC on the Tibetan Plateau grasslands was almost two time higher than that in the Inner Mongolian grasslands (11.30 vs. 6.08 mg/g) (Fig. 3C).

For all 151 species, leaf phenolics varied from 0.11 to 190.55 mg/g with an average of 24.69 mg/g, and UVAC varied from 2.53 to 30.35 mg/g with an average of 10.60 mg/g. In the Inner Mongolian grasslands, leaf phenolics and UVAC ranged between 0.11–116.54 and 2.61–27.82 mg/g with an average of 15.28 and 8.70 mg/g, respectively. By contrast, leaf phenolics and UVAC varied between 1.02–190.55 and 2.53–30.35 mg/g with an average of 30.43 and 11.69 mg/g on the Tibetan Plateau grasslands, respectively. Similarly, at the species level, leaf phenolics and UVAC show markedly differences between Inner Mongolian and Tibetan Plateau grasslands. The mean of leaf phenolics and UVAC on the Tibetan Plateau grasslands was almost two time higher than those in the Inner Mongolian grassland (30.43 vs. 15.28 mg/g and 11.69 vs. 8.70 mg/g), which was not affected by phylogenetic association (λ = 0.000, Fig. 3B and D).

**Figure 4:**
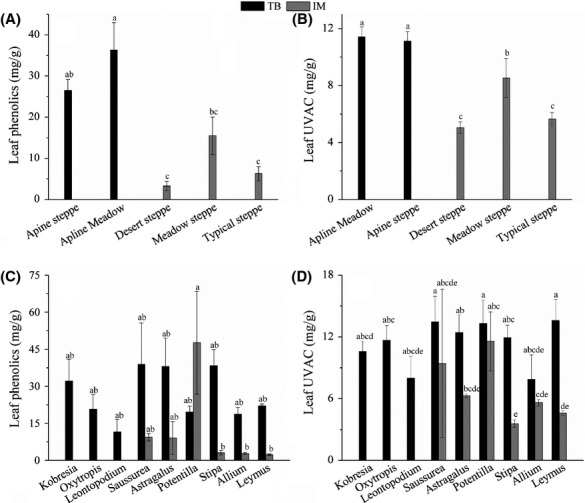
Leaf phenolics and UV-absorbing compounds (UVAC) for five vegetable types and nine common genera (with more than 10 species) at species level. The values indicate means ± SE, with different letters above the values indicating significant difference (*P* < 0.05) among genera and, between Inner Mongolia and Tibetan Plateau grasslands tested using one-way ANOVA with a Duncan post hoc test.

For vegetative type, leaf phenolics and UVAC in alpine meadow and steppe dominant in the Tibetan Plateau grasslands were significantly higher than those in typical, meadow, and desert steppe common in the Inner Mongolian grasslands (Fig. 4A and B). At the genus level, they were also significantly lower in *Leymus* and *Allium* dominant in the Inner Mongolian grasslands than those in other 7 genera dominant in the Tibetan Plateau grasslands (Table S3), and within the same genus such as *Saussurea*, *Astragalus*, *Stipa*, *Allium,* and *Leymus* except *Potentilla*, they were higher on the Tibetan Plateau than those in Inner Mongolian grasslands despite not significantly (Fig. 4C and D). For functional groups, leaf phenolics and UVAC in herb and grass were higher than those in shrub, but not significantly so (Table S3).

### UVR was the primary driver of leaf phenolics

**Figure 5:**
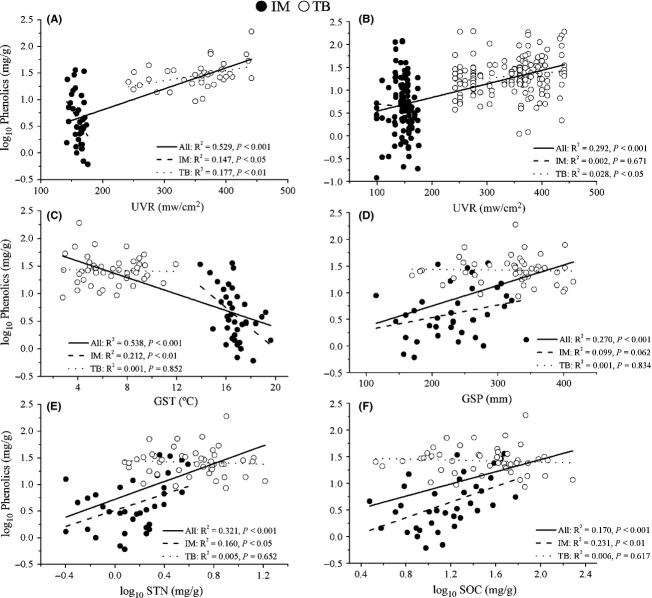
Relationships between leaf phenolics and environmental factors: (A, B) UVR at the site and species-by-site levels, respectively; (C) GST, (D) GSP, (E) STN, (F) SOC at the site level. R-squared (R^2^) and *P*-values for overall and contrasting regions (IM, Inner Mongolia; TB, Tibetan Plateau) were estimated at the site level using linear regression models.

Across all grasslands, a significant positive relationship between leaf phenolics and UVR was found at both the site level (Fig. 5A) and species-by-site level (Fig. 5B). However, this relationship differed within the Tibetan Plateau and Inner Mongolian grasslands. For instance, leaf phenolics significantly increased with UVR on the Tibetan Plateau grasslands at both the site level and species-by-site level, whereas it decreased markedly with UVR at the site level, and even had no relation with UVR at species-by-site level in the Inner Mongolian grasslands, where the varying range of UVR across sites was quite narrow (142–176 mW/cm^2^) (Fig. 5A and B). Additionally, leaf phenolics were significantly negatively correlated with GST (Fig. 5C) while positively correlated with GSP (Fig. 5D), STN (Fig 5E), and SOC (Fig. 5F) at the site level. However, the strength of these correlations became weaker in the Inner Mongolia grasslands, even disappeared on the Tibetan Plateau grasslands (Fig 5C–F).

**Figure 6:**
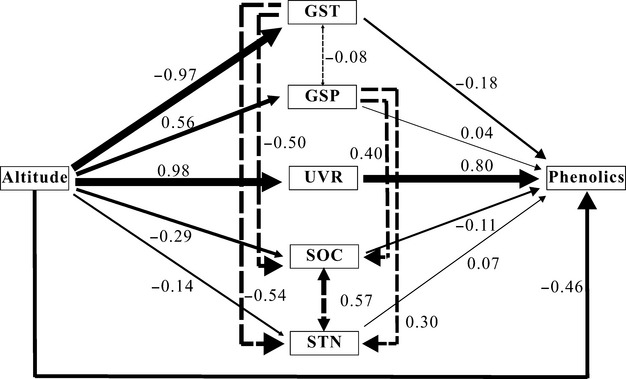
Best-fit structural equation model combining Altitude, GST, GSP, UVR, SOC, and STN across all sites (χ^2^ = 6.04, df = 4, *P* = 0.20). The model shown (M5 in Table 1) includes the correlations among environmental factors. The width of arrows indicates the strength of the causal effect. Values give the standardized coefficients for the relationship between “upstream” and “downstream” variables. See Fig. 2 for the full set of models.

Structural equation modeling analysis showed that UV radiation was the strongest driver of leaf phenolic concentrations at the site level (Fig. 6). The SEM with altitude as the primary driver for all climatic and site characteristics, and where the covariances between climate and soil factors and UVR were included was the best fitting of the six alternative structures evaluated (Table 1).

**Table 1:** Results of structural equation modeling comparisons for the effects of altitude, GST, GSP, UVR, SOC, and STN as predictors of the environmental effect on leaf total phenolics across 84 sites from Inner Mongolia and Tibetan Plateau grasslands. Model M5 is the final model presented in Fig. 6; see the Supporting Information for the other models.

Model	No. sites	χ^2^	df	*P*	BIC	RMSEA	CFI
M1	84	15.24	3	0.002	1.951	0.222	0.979
M2	84	13.17	1	0.000	8.744	0.383	0.959
M3	84	208.09	10	0.000	163.784	0.489	0.752
M4	84	202.05	6	0.000	175.468	0.627	0.624
M5	84	6.04	4	0.196	−11.685	0.078	0.997
M6	84	−6.03E–13	0	1.000	0.000	NA	NA

### Leaf phenolics covaried with other leaf traits

**Figure 7:**
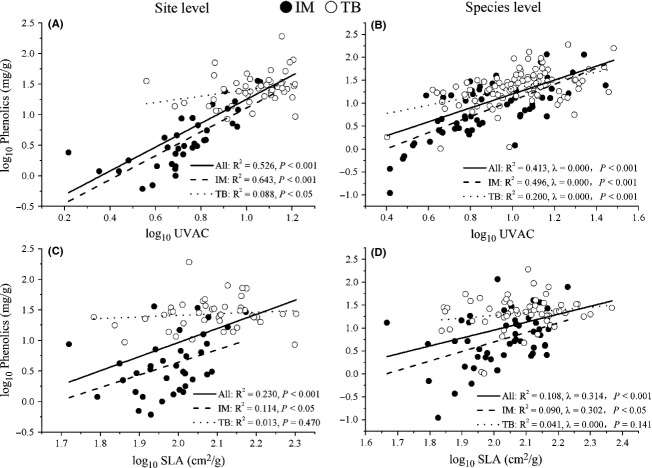
Relationships between leaf phenolics and other leaf traits: (A, B) ultraviolet-absorbing compounds (UVAC), (C, D) specific leaf area (SLA) at the site and species level, respectively. R-squared (R^2^) and *P*-values for overall and contrasting regions (IM, Inner Mongolia; TB, Tibetan Plateau) were estimated at the site level using linear regression models, and at the species level using PGLS regression; Lambda (λ) values indicate phylogenetic independence of traits.

**Figure 8:**
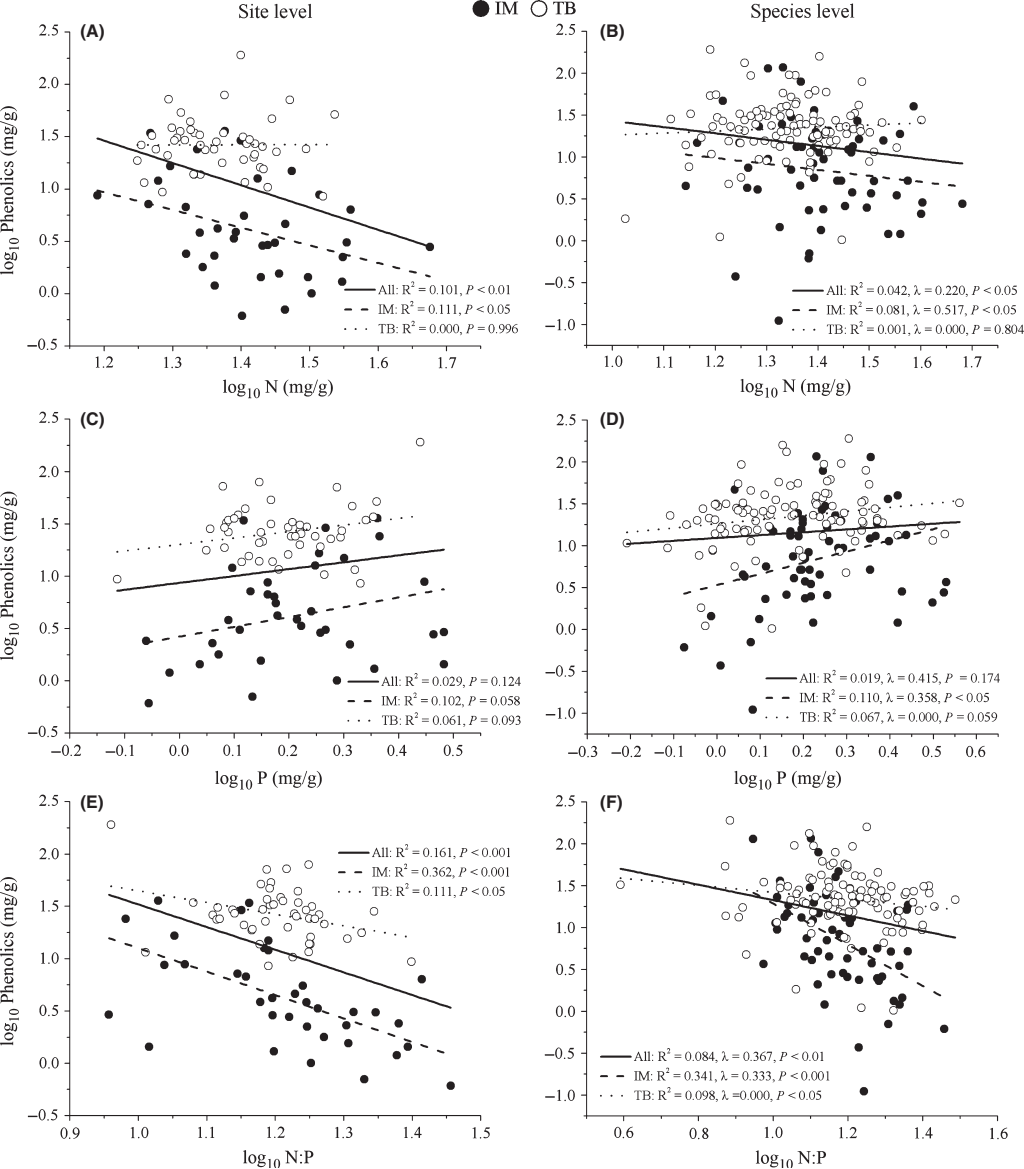
Relationships between leaf phenolics and leaf nutrient elements: (A, B) leaf N, (C, D) leaf P and, (E, F) N: P ratio at the site and species level, respectively. R-squared (R^2^) and *P*-values for overall and contrasting regions (IM, Inner Mongolia; TB, Tibetan Plateau) were estimated at the site level using linear regression models, and at the species level using PGLS regression; Lambda (λ) values indicate phylogenetic independence of traits.

Linear and PGLS regression analyses showed that leaf phenolics were strongly and positively correlated with UV-absorbing compounds (UVAC) at both the site and species level. Single and Multiple regressions showed that the strength of the correlations were strongest in the Inner Mongolian grasslands (Fig. 7A and B, Table S4), and these relationships were not influenced by the plant relatedness (all λ = 0.000). Leaf phenolics were also significantly and positively correlated with SLA (Fig. 7C and D), but negatively correlated with leaf N (Fig. 8A and B) and N: P ratios (Fig. 8E and F), and had no relationship with leaf P (Fig. 8C and D) at either the site or species level. The relationships between leaf phenolics and other leaf traits were independent of the phylogeny (all λ = 0.000) in the Tibetan Plateau grasslands, conversely they were much more affected by the phylogenetic association (λ = 0.302–0.517) in the Inner Mongolian grasslands. Considering the contrasting regions, the correlations between leaf phenolics and other leaf traits became significantly stepper in the Inner Mongolian grasslands (Table 2, Figs. 7, 8).

**Table 2:** Bivariate trait comparisons of grassland plants sampled in Inner Mongolia and Tibetan Plateau by phylogenetic generalized least-squares regressions at species level. Measure of phylogenetic correlation, λ. Coefficients and standard errors of log_10_-transformed traits.

Traits	λ	Inner Mongolia		Tibetan Plateau	
	
		Intercept (± SE)	Slope (± SE)	Intercept (± SE)	Slope (± SE)
Phenolics-UVAC	0.000	−0.74 ± 0.23***	1.74 ± 0.26***	0.34 ± 0.28***	1.00 ± 0.28***
Phenolics-SLA	0.042	−2.03 ± 1.32	1.38 ± 0.64*	0.01 ± 0.88**	0.63 ± 0.42*
Phenolics-N	0.179	2.90 ± 1.07*	−1.49 ± 0.73**	1.51 ± 0.73**	−0.14 ± 0.55
Phenolics-P	0.330	0.43 ± 0.25	1.50 ± 0.62*	1.23 ± 0.07***	0.63 ± 0.33***
Phenolics-N:P	0.135	4.17 ± 0.71***	−2.85 ± 0.58***	2.40 ± 0.45***	−0.90 ± 0.38**

Significance of coefficients (*t-*tests) indicated: ***P < 0.001; **P < 0.01; *P < 0.05. Significance of Inner Mongolia intercepts and slopes is tested for heterogeneity from zero. Tibetan Plateau intercepts and slopes are tested for heterogeneity from Inner Mongolia intercepts and slopes, respectively.

## Discussion

### UV radiation (UVR) as a primary diver affecting plant phenolics

Previous studies of the role of phenolics have largely focused on a specific phenolic component within a few given species in a controlled setting, for example, the role of flavonoids, phenolic acids, and condensed tannins in grass, woody, moss, or other species in response to ultraviolet-B radiation (Hofmann et al. 2000; de la Rosa et al. 2001; Turtola et al. 2005; Dunn and Robinson 2006; Martz et al. 2007; Thines et al. 2007). However, in the field, it is challenging to distinguish the effects of herbivory and UVR on the production of leaf phenolics, much less the role of particular phenolic compounds. As shown in other studies, the variation in the concentrations of leaf phenolics in natural conditions reflects both the responses of an individual plants to environmental stressors (e.g., UVR or grazing) as well as species-level adaptations to such stressors (Rozema et al. 1997; Jansen et al. 1998). To this end, we minimized the effects of disturbances (e.g., grazing) on generating leaf phenolics by sampling plants from undisturbed grasslands. In this case, our results suggested that the variation of leaf phenolics mainly resulted from protection against UVR, rather than grazing by herbivores. We found that leaf phenolics in plants from the high-elevation Tibetan Plateau grasslands, where UVR is relatively higher, were significantly higher than that in plants growing in the Inner Mongolian grasslands with lower and narrower range of UVR for species, genera, and vegetative types at site level. Furthermore, the difference between Tibetan Plateau and Inner Mongolian grasslands was independent of the phylogeny. Importantly, across all our study sites, leaf phenolics significantly increased with UVR, and SEM analysis showed that UVR is the primary factor driving the variation in leaf phenolics. Our findings were consistent with many above-mentioned studies under controlled experiments showing that high light and UVB resulted in the accumulation of leaf phenolics. However, other studies showed that total leaf phenolics did not exhibit apparent changes following short-term manipulated UVR (Rousseaux et al. 1998; Salt et al. 1998; Levizou and Manetas 2001). We suspect that this may be because short-term manipulation of UVR could activate induced resistance (Roberts and Paul 2006), which mainly change the ratios of different phenolic types, but not enough to increase the constitutive resistance (Yeoman and Yeoman 1996) under the long-term selection, adaptation, and evolution in environmental stresses (e.g., UVR in our study) (Jormalainen and Honkanen 2004).

In addition, leaf UVAC also showed a similar trend with leaf phenolics between Tibetan Plateau and Inner Mongolian grasslands. This result is consistent with the view that one of the most consistent responses of plants to elevated UVB exposure is the synthesis of UV-absorbing compounds in foliage (Searles et al. 2001; Clarke and Robinson 2008; Newsham and Robinson 2009). Further, we found that leaf phenolics significantly increased with UVAC at both the species and site level across all grasslands. This close relationship was not affected by the phylogenetic association, further supporting that leaf phenolics are mainly caused by UVR. Further evidence of the photo-protective role of phenolics comes from observations that the ratio of the “efficient antioxidant” phenolics, such as quercetin or luteolin glycosides, to the “poor antioxidant” kaempferol or apigenin glycosides increases for plants exposed high levels of UVB and sunlight irradiation (Markham et al. 1998; Agati et al. 2009). Furthermore, some studies have also reported that leaf phenolics accumulate not only in the vacuoles and cell walls of epidermal cell and in trichomes (Wollenweber and Dietz 1981; Strack et al. 1988; Hutzler et al. 1998; Tattini et al. 2007), but also in the vacuoles of mesophyll cell (Kytridis and Manetas 2006; Agati et al. 2009) and in chloroplasts (Agati et al. 2007) following sunlight exposure. These findings all demonstrate that phenolic function protect leaves from photo-oxidative damage via acting as antioxidants, not only by screening the short-wavelength solar UV radiation (Close and McArthur 2002; Ryan et al. 2002; Agati et al. 2009, 2011, 2012; Agati and Tattini 2010). Consequently, higher leaf phenolics and UVAC could facilitate plants' adaptation under the strong light and UV radiation conditions, particularly in the high-altitude Tibetan Plateau.

### Difference effects of climatic and soil factors on leaf phenolics between Tibetan Plateau and Inner Mongolian grasslands

There was a significant positive relationship between leaf phenolics and UVR, and when accounting for the complex correlations between site characteristics including UVR and leaf phenolic concentrations using SEM, we found that UVR was by far the strongest predictor of total leaf phenolics across our all study sites. However, the effects of climatic and soil factors on leaf phenolics differed greatly between Tibetan Plateau and Inner Mongolian grasslands.

The biome-level difference, where there is little variation in leaf phenolics for plants on the Tibetan Plateau despite considerable variation in UV radiation, may result from the strong selection pressure exerted on plants by the physical environment on the Tibetan Plateau, with high UV radiation and severe temperatures. Hence, the phenolic contents of plants growing on the Tibetan Plateau converge on a relatively narrow range of leaf phenolics due to this strong environmental filter. Yet even despite such convergence, total leaf phenolics were positively and significantly correlated only with UVR even within just the Tibetan Plateau (Fig. 5A and B). In contrast, the more moderate physical environment (e.g., low UV radiation and high temperature) on the Inner Mongolian grasslands exerts smaller selection pressures. This allows a wider range of leaf phenolics on the Inner Mongolian grasslands at both the site and species levels. At the site level in the Inner Mongolian grasslands, leaf phenolics were significantly affected by temperature, soil nitrogen, and soil carbon, and not only UV radiation. Consistent with our results, several studies documented that the declining temperature leads to a pronounced increase in phenolics (Kuokkanen et al. 2001; Pennycooke et al. 2005; Zvereva and Kozlov 2006; Albert et al. 2009); and Turtola et al. (2005) showed that the decreasing water availability reduced the total concentration of salicylates and phenolic acids in *Salix myrsinifolia* plantlets. For nutrient availability, there are conflicting results. de la Rosa et al. (2001) showed that nutrient addition reduced the concentrations of phenolics in silver birch seedling, whereas Sundqvist et al. (2012) suggested that total phenolic content in litters was often higher for species that dominate on more fertile soil in Swedish subarctic tundra, which was in line with our results. The variability in these environmental factors thus might mask the UV effect in the Inner Mongolian grasslands. Alternatively, the low UV doses (only 161 mW/cm^2^ on average) in the Inner Mongolian grasslands may be insufficient to trigger the responses of the phenylalanine ammonia-lyase (PAL) pathway. de la Rosa et al. (2001) demonstrated that individual phenolics in silver birch were a dose-dependent response to UBV radiation.

### Leaf phenolics covary with other leaf functional traits

It is well known that plants have to balance their fixed biomass among growth, reproduction, and defense (Bazzaz and Grace 1997), which can be translated into trade-offs among plant functional traits from individual to species and community level (Ackerly et al. 2000; Suding et al. 2003; Violle et al. 2007). However, very few studies tested the relationships between leaf phenolics and other leaf functional traits, such as SLA and leaf N, P, N:P ratio, which they can indicate plant ecological strategy in trade-offs of capturing limited light and exploiting soil resource (Violle et al. 2007). Although UVR markedly influenced plant growth and reproduction by generating mass of UVAC and phenolics (Weinig et al. 2004), we did not know whether the production of leaf phenolics would incur the costs of other plant functional traits. If so, and then what effect may be produced for community attributes and ecosystem functions.

In our previous research, we addressed that the patterns of key functional traits, for example, SLA and stoichiometry of leaf N: C and N: P (He et al. 2006a,b, 2008) and explored the underlying determinant factors, for example, the relative important of taxonomic, phylogenetic, and environmental changes in determining the trade-offs in leaf productivity–persistence in Chinese grasslands (He et al. 2009). In this study, we predicted that leaf phenolics should be related to these leaf functional traits, for example, SLA and leaf N, P, N: P ratio due to trade-offs between growth and defense. We found leaf phenolics tended to increase with SLA and decrease with leaf N and N: P ratio, particularly in the Inner Mongolian grasslands. Our finding about the positive correlation between leaf phenolics and SLA is contrary to the result of Peñuelas et al. (2011). Despite no relationship between leaf phenolics and leaf P, the significant negative relationships between leaf phenolics and leaf N and N: P are well in agreement with previous results at the across-species level (Wright et al. 2010; Peñuelas et al. 2011). These results suggested that plants with larger SLA and low N: P could generate more leaf phenolics because larger area was exposed to UVR and slow growth (meaning longer exposure to UVR) (Vanni et al. 2002; Elser et al. 2003). Separating cause and effect in the relationship between leaf phenolics and leaf characteristics in these grassland systems will require controlled experiments. Our results indicated that the production of leaf phenolics may also influence on community attributes and vegetation processes via changes in leaf functional traits. Therefore, future studies are urgently needed to clarify the important consequences of plant secondary metabolites for soil nutrition cycle and further ecosystem processes.

## Conclusion

Our results indicated that leaf phenolics in the Tibetan Plateau grasslands with higher UV radiation were significantly higher than that in the Inner Mongolian grasslands with lower UV radiation from the site-, species-, genus- to vegetative type level. Most importantly, we found that UV radiation was the strongest climatic factor driving the variation of leaf phenolics across Chinese grasslands, suggesting that higher leaf phenolics could facilitate plants' adaptation under the strong light and UV radiation conditions, particularly in the high-altitude Tibetan Plateau. Our results also indicate that leaf phenolics may influence on vegetation attributes and indirectly on ecosystem processes by covarying with other leaf functional traits.

## Supporting Information

**Table S1.** Description of 84 sites across the Tibetan Plateau (TB) and Inner Mongolian (IM) grasslands where leaf traits measurements were taken.

**Table S2.** Data of leaf traits for 151 sampled species from the Tibetan Plateau (TB) and Inner Mongolian (IM), and both regions (TB/IM).

**Table S3.** Leaf phenolics, UV-absorbing compounds (UVAC), and leaf traits, including specific leaf area (SLA), leaf N, P, and N:P ratio, for 5 vegetable types, 3 functional groups at site level and 9 common genera (with more than 10 species) at species-by-site level.

**Table S4.** The correlation coefficients of phenolics and other leaf traits including SLA, leaf C, N, leaf C:N, and leaf UVAC using the multiple regression PGLS under considering the regional differences.

**Figure S1.** Phylogeny hypothesis for 151 Tibetan Plateau and Inner Mongolian grassland plants used in trait analyses.
