# Supplementary material for: UV radiation is the primary factor driving the variation in leaf phenolics across Chinese grasslands
Source: Ecol Evol. 2013 Oct 29;3(14):4696–710. doi: 10.1002/ece3.862 (PMC3867905; doi:10.1002/ece3.862)

**Chen *et al*. UV radiation is the primary factor driving the variation in leaf phenolics across Chinese grasslands**

**Supporting Information**

**Table S1.** Description of 84 sites across the Tibetan Plateau (TB) and Inner Mongolian (IM) grasslands where leaf traits measurements were taken. Data for latitude, longitude and altitude were obtained with Magellan GPS Field PROV (Magellan System Corporation, San Dimas, CA, USA). Growing season temperature (GST) and growing season precipitation (GSP) were compiled from the 1950-2000 temperature/precipitation records of a global climate database .

| **Site** | **Longitude (ºE)** | **Latitude (ºN)** | **Altitude (m)** | **GST (ºC)** | **GSP**  **(mm)** | **UVR**  **(mw cm^-2^)** | **Vegetation type** |
| --- | --- | --- | --- | --- | --- | --- | --- |
| IM01 | 116.74 | 43.60 | 1209 | 16.20 | 208 | 174 | Typical steppe |
| IM02 | 116.33 | 45.02 | 847 | 14.94 | 206 | 163 | Typical steppe |
| IM03 | 116.29 | 43.89 | 1213 | 16.40 | 229 | 168 | Typical steppe |
| IM04 | 115.82 | 44.64 | 965 | 17.30 | 232 | 168 | Typical steppe |
| IM05 | 116.33 | 45.02 | 847 | 17.60 | 232 | 169 | Typical steppe |
| IM06 | 117.59 | 45.73 | 920 | 16.70 | 262 | 169 | Typical steppe |
| IM07 | 118.78 | 47.87 | 741 | 16.20 | 305 | 152 | Typical steppe |
| IM08 | 118.47 | 48.27 | 702 | 16.50 | 299 | 150 | Typical steppe |
| IM09 | 118.87 | 48.84 | 719 | 16.10 | 284 | 145 | Typical steppe |
| IM10 | 120.12 | 49.35 | 668 | 15.60 | 321 | 145 | Meadow steppe |
| IM11 | 120.05 | 49.33 | 625 | 16.00 | 313 | 148 | Meadow steppe |
| IM12 | 119.71 | 49.08 | 633 | 16.50 | 285 | 156 | Typical steppe |
| IM13 | 119.99 | 49.89 | 723 | 14.70 | 261 | 143 | Typical steppe |
| IM14 | 120.00 | 50.19 | 555 | 15.30 | 228 | 156 | Meadow steppe |
| IM15 | 119.02 | 49.96 | 571 | 16.30 | 185 | 152 | Typical steppe |
| IM16 | 117.57 | 49.55 | 658 | 16.50 | 209 | 142 | Typical steppe |
| IM17 | 116.99 | 49.10 | 630 | 16.9 | 229 | 156 | Typical steppe |
| IM18 | 116.23 | 48.77 | 800 | 16.70 | 245 | 161 | Typical steppe |
| IM19 | 116.95 | 48.58 | 553 | 17.60 | 263 | 158 | Typical steppe |
| IM20 | 117.84 | 48.69 | 571 | 17.20 | 278 | 158 | Typical steppe |
| IM21 | 117.80 | 49.12 | 587 | 17.10 | 343 | 157 | Typical steppe |
| IM22 | 118.24 | 49.46 | 625 | 16.50 | 310 | 158 | Typical steppe |
| IM23 | 119.34 | 49.304 | 586 | 16.60 | 254 | 154 | Meadow steppe |
| IM24 | 119.76 | 46.44 | 1080 | 13.90 | 209 | 170 | Meadow steppe |
| IM25 | 119.33 | 45.845 | 905 | 15.80 | 261 | 166 | Meadow steppe |
| IM26 | 118.23 | 45.75 | 870 | 16.70 | 266 | 167 | Typical steppe |
| IM27 | 116.98 | 45.42 | 842 | 17.50 | 239 | 161 | Typical steppe |
| IM28 | 117.46 | 44.77 | 1048 | 16.00 | 240 | 166 | Meadow steppe |
| IM29 | 117.36 | 44.51 | 1058 | 16.10 | 196 | 166 | Typical steppe |
| IM30 | 116.33 | 44.13 | 1096 | 16.90 | 177 | 167 | Typical steppe |
| IM31 | 114.85 | 43.99 | 1152 | 16.90 | 157 | 169 | Desert steppe |
| IM32 | 116.33 | 44.13 | 1157 | 16.90 | 115 | 170 | Desert steppe |
| IM33 | 113.45 | 43.82 | 955 | 18.40 | 145 | 164 | Desert steppe |
| IM34 | 112.18 | 43.638 | 955 | 19.60 | 160 | 165 | Desert steppe |
| IM35 | 112.57 | 42.88 | 1102 | 19.20 | 173 | 171 | Desert steppe |
| IM36 | 112.38 | 42.36 | 1216 | 18.90 | 160 | 163 | Desert steppe |
| IM37 | 111.83 | 41.79 | 1422 | 18.20 | 173 | 176 | Desert steppe |
| QZ01 | 101.48 | 36.37 | 3454 | 7.33 | 339 | 309 | Alpine meadow |
| QZ02 | 101.30 | 35.80 | 3302 | 8.98 | 333 | 275 | Alpine meadow |
| QZ03 | 101.17 | 35.78 | 3263 | 9.37 | 320 | − | Alpine steppe |
| QZ04 | 101.08 | 35.56 | 3416 | 8.50 | 333 | − | Alpine steppe |
| QZ05 | 101.09 | 35.63 | 3643 | 8.98 | 340 | 305 | Alpine meadow |
| QZ06 | 100.97 | 35.41 | 3517 | 7.99 | 343 | 332 | Alpine meadow |
| QZ07 | 100.25 | 34.24 | 4282 | 3.96 | 402 | − | Alpine meadow |
| QZ08 | 99.88 | 33.96 | 4053 | 6.06 | 390 | 360 | Alpine meadow |
| QZ09 | 100.07 | 34.01 | 4189 | 8.50 | 336 | 359 | Alpine steppe |
| QZ10 | 99.87 | 33.93 | 4099 | 6.84 | 351 | 361 | Alpine meadow |
| QZ11 | 99.83 | 33.94 | 4156 | 5.38 | 402 | 350 | Alpine meadow |
| QZ12 | 99.80 | 33.80 | 3972 | 7.99 | 346 | 345 | Alpine meadow |
| QZ13 | 99.40 | 34.06 | 4231 | 5.05 | 389 | − | Alpine meadow |
| QZ14 | 98.21 | 34.92 | 4267 | 4.96 | 326 | 375 | Alpine meadow |
| QZ15 | 98.23 | 34.89 | 4224 | 5.27 | 325 | 365 | Alpine meadow |
| QZ16 | 98.00 | 34.57 | 4312 | 3.96 | 414 | − | Alpine meadow |
| QZ17 | 97.88 | 34.28 | 4667 | 2.84 | 364 | 410 | Alpine meadow |
| QZ18 | 96.28 | 33.32 | 4506 | 5.48 | 333 | 396 | Alpine meadow |
| QZ19 | 95.80 | 34.01 | 4201 | 7.15 | 274 | 350 | Alpine steppe |
| QZ20 | 95.80 | 34.15 | 4238 | 5.77 | 399 | 362 | Alpine steppe |
| QZ21 | 96.16 | 34.10 | 4415 | 5.38 | 402 | 375 | Alpine meadow |
| QZ22 | 97.60 | 34.06 | 4700 | 2.97 | 365 | 436 | Alpine meadow |
| QZ23 | 99.01 | 35.29 | 4217 | 4.47 | 336 | 370 | Alpine steppe |
| QZ24 | 100.25 | 36.01 | 3109 | 10.92 | 274 | 250 | Alpine steppe |
| QZ25 | 100.51 | 36.17 | 2925 | 11.93 | 264 | − | Alpine steppe |
| QZ26 | 100.74 | 36.36 | 3233 | 9.43 | 287 | 251 | Alpine steppe |
| QZ27 | 101.09 | 36.44 | 3486 | 7.32 | 314 | 315 | Alpine meadow |
| QZ28 | 100.86 | 36.95 | 3130 | 9.62 | 265 | 242 | Alpine steppe |
| QZ29 | 99.98 | 37.26 | 3215 | 9.39 | 233 | 260 | Alpine steppe |
| QZ30 | 98.99 | 37.28 | 3437 | 8.48 | 216 | 288 | Alpine steppe |
| QZ31 | 94.25 | 35.74 | 4222 | 6.70 | 170 | 360 | Alpine steppe |
| QZ32 | 93.74 | 35.52 | 4564 | 4.80 | 188 | 390 | Alpine steppe |
| QZ33 | 93.04 | 35.17 | 4682 | 4.31 | 182 | 441 | Alpine steppe |
| QZ34 | 92.89 | 34.72 | 4801 | 3.76 | 249 | 433 | Alpine meadow |
| QZ35 | 92.35 | 33.99 | 4654 | 4.94 | 248 | 393 | Alpine steppe |
| QZ36 | 91.72 | 32.18 | 4903 | 4.12 | 327 | − | Alpine meadow |
| QZ37 | 91.72 | 32.18 | 4902 | 4.12 | 327 | 441 | Alpine meadow |
| QZ38 | 92.02 | 31.45 | 4494 | 7.94 | 341 | 375 | Alpine meadow |
| QZ39 | 92.02 | 31.44 | 4486 | 7.94 | 341 | 380 | Alpine meadow |
| QZ40 | 92.62 | 31.77 | 4605 | 5.89 | 361 | 406 | Alpine meadow |
| QZ41 | 92.41 | 31.69 | 4596 | 6.00 | 355 | − | Alpine meadow |
| QZ42 | 91.66 | 30.94 | 4756 | 5.45 | 371 | − | Alpine steppe |
| QZ43 | 91.45 | 30.56 | 4506 | 7.32 | 359 | 400 | Alpine meadow |
| QZ44 | 90.80 | 30.31 | 4324 | 8.81 | 326 | 370 | Alpine steppe |
| QZ45 | 91.86 | 32.58 | 5105 | 2.77 | 331 | − | Alpine meadow |
| QZ46 | 92.61 | 34.37 | 4656 | 4.78 | 241 | 396 | Alpine steppe |
| QZ47 | 99.67 | 36.78 | 3391 | 8.72 | 251 | 275 | Alpine meadow |

**Table S2.** Data of leaf traits for 151 sampled species from the Tibetan Plateau (TB) and Inner Mongolian (IM), and both regions (TB/IM). Abbreviations: SLA, species leaf area (cm^2^ g^-1^); C, leaf carbon concentration (mg g^-1^); N, leaf N concentration (mg g^-1^); P, leaf P concentration (mg g^-1^); Phenolics, leaf total phenolics (mg g^-1^); UVAC, ultraviolet absorbing compounds (mg g^-1^); FG, functional group: H, herb, including annuals, biennials and perennials forbs; S, shrub, including deciduous shrubs and evergreen shrubs; G, grass, including graminoids and sedges.

| **Species** | **Regions** | **FG** | **SLA** | **C** | **N** | **P** | **C:N** | **N:P** | **Phenolics** | **UVAC** |
| --- | --- | --- | --- | --- | --- | --- | --- | --- | --- | --- |
| *Acomastylis elata* | TB | H | NA | 468.60 | 24.80 | 1.83 | 18.90 | 13.55 | 61.66 | 27.34 |
| *Aconitum pendulum* | TB | H | NA | 458.10 | 29.10 | 1.15 | 15.74 | 25.30 | 30.90 | 11.31 |
| *Adenophora paniculata* | IM | H | 113.28 | 443.30 | 22.30 | 1.78 | 19.88 | 12.56 | 29.72 | 6.29 |
| *Agropyron crastatum* | IM/TB | G | 88.02 | 468.45 | 21.38 | 1.25 | 23.61 | 17.31 | 8.76 | 5.01 |
| *Agropyron mongolicum* | IM | G | 107.22 | 448.3 | 28.40 | 1.80 | 15.79 | 15.81 | 2.57 | 4.47 |
| *Agrostis alba* | IM | G | 139.45 | 448.00 | 22.30 | 1.60 | 20.09 | 13.93 | 6.99 | 11.48 |
| *Ajania khartensis* | TB | H | 68.66 | 468.80 | 21.80 | 0.98 | 21.50 | 22.24 | 8.71 | 7.79 |
| *Ajania pallasiana* | TB | H | 130.72 | 445.00 | 15.70 | 0.78 | 28.34 | 20.13 | 22.91 | 8.99 |
| *Allium anisopodium* | IM | H | 102.52 | 427.30 | 34.45 | 2.62 | 12.43 | 13.73 | 1.20 | 3.36 |
| *Allium_carolinianum* | TB | H | NA | 435.00 | 25.20 | 1.32 | 17.26 | 19.09 | 16.98 | 4.68 |
| *Allium_cyaneum* | TB | H | NA | 463.25 | 16.85 | 1.16 | 27.69 | 14.98 | 15.37 | 6.00 |
| *Allium mongolicum* | IM | H | 97.79 | 416.67 | 39.90 | 3.15 | 10.49 | 13.17 | 2.08 | 6.23 |
| *Allium polyrhizum* | IM | H | 95.22 | 442.39 | 40.11 | 2.68 | 11.46 | 15.40 | 2.84 | 5.57 |
| *Allium_ramosum* | IM | H | 131.97 | 441.50 | 48.00 | 3.35 | 9.20 | 14.32 | 2.76 | 5.33 |
| *Allium senescens* | IM | H | 82.68 | 431.30 | 27.90 | 2.26 | 15.47 | 13.20 | 5.16 | 6.35 |
| *Allium tanguticum* | TB | H | NA | 435.20 | 16.40 | 1.18 | 26.54 | 13.9 | 26.92 | 14.79 |
| *Allium tenuissimum* | IM | H | 132.99 | 462.10 | 29.30 | 1.60 | 15.77 | 18.36 | 3.73 | 7.21 |
| *Anaphalis hancockii* | TB | H | NA | 459.15 | 30.15 | 1.81 | 15.61 | 16.58 | 41.62 | 10.43 |
| *Anaphalis sinica* | TB | H | 190.38 | 453.50 | 20.85 | 0.85 | 24.26 | 27.03 | 17.79 | 7.97 |
| *Androsace mariae* | TB | H | 91.41 | 452.00 | 16.20 | 0.94 | 27.90 | 17.23 | 1.10 | 9.68 |
| *Artemisia desertorum* | TB | S | NA | 450.75 | 18.85 | 1.18 | 24.15 | 16.38 | 15.19 | 8.53 |
| *Artemisia frigida* | IM/TB | H | 114.11 | 455.33 | 18.45 | 1.34 | 25.80 | 14.79 | 26.68 | 8.11 |
| *Artemisia tanacetifolia* | IM | H | 107.48 | 464.70 | 21.40 | 1.54 | 21.71 | 13.88 | 24.40 | 27.82 |
| *Artemisia tangutica* | TB | H | NA | 453.30 | 23.10 | 1.44 | 19.62 | 16.04 | 28.84 | 9.48 |
| *Asparagus gobicus* | IM | S | 109.07 | 461.90 | 29.20 | 1.82 | 15.82 | 16.04 | 11.30 | 5.99 |
| *Aster tataricus* | TB | H | 123.92 | 437.50 | 16.85 | 1.99 | 26.01 | 8.47 | 4.74 | 8.51 |
| *Astragalus adsurgens* | IM | H | 130.45 | 429.30 | 31.30 | 1.65 | 13.72 | 18.99 | 2.46 | 6.11 |
| *Astragalus confertus* | TB | H | 93.76 | 469.00 | 25.25 | 3.37 | 19.20 | 7.47 | 13.65 | 17.61 |
| *Astragalus fenzelianus* | TB | H | 155.74 | 478.40 | 17.00 | 1.31 | 28.14 | 12.98 | 22.91 | 5.38 |
| *Astragalus licentianus* | TB | H | 148.21 | 461.40 | 26.00 | 2.42 | 17.75 | 11.48 | 18.70 | 7.60 |
| *Astragalus mattam* | TB | H | NA | 478.40 | 18.60 | 1.14 | 25.72 | 16.32 | 93.33 | 11.88 |
| *Astragalus polycladus* | TB | H | 110.21 | 405.83 | 15.50 | 2.25 | 26.53 | 7.43 | 53.87 | 14.74 |
| *Astragalus uliginosus* | IM | H | NA | 443.20 | 33.70 | 1.57 | 13.15 | 21.51 | 15.71 | 6.49 |
| *Belamcanda chinensis* | IM | H | 46.37 | 436.95 | 23.40 | 2.33 | 19.43 | 11.90 | 13.05 | 10.31 |
| *Bromus inermis* | IM | G | 147.66 | 451.20 | 20.10 | 1.96 | 22.45 | 10.26 | 9.39 | 12.44 |
| *Bupleurum chinense* | IM | H | 132.79 | 452.30 | 24.60 | 1.63 | 18.39 | 15.06 | 8.33 | 12.45 |
| *Bupleurum commelynoideum* | TB | H | 130.60 | 478.15 | 22.90 | 1.23 | 21.13 | 18.77 | 19.60 | 4.17 |
| *Caragana sinica* | IM | S | NA | 472.3 | 36.30 | 1.58 | 13.01 | 22.99 | 18.66 | 6.42 |
| *Caragana stenophylla* | IM | S | 102.23 | 469.10 | 36.40 | 1.67 | 12.89 | 21.84 | 1.20 | 10.29 |
| *Carex alrofusca* | TB | G | 107.33 | 434.40 | 19.97 | 0.98 | 23.24 | 20.12 | 8.93 | 5.56 |
| *Carex korshinski* | TB | G | 127.61 | 390.40 | 15.50 | 2.02 | 25.19 | 7.67 | 190.55 | 18.42 |
| *Carex moorcroftii* | TB | G | 117.43 | 433.10 | 23.20 | 1.22 | 18.68 | 22.54 | 24.57 | 9.49 |
| *Carex pediformis* | IM | G | 116.26 | 457.43 | 17.67 | 1.48 | 25.95 | 13.25 | 14.8 | 5.11 |
| *Ceratoides latens* | TB | S | NA | 421.20 | 17.80 | 1.10 | 23.66 | 16.18 | 5.62 | 19.75 |
| *Cleistogenes songorica* | IM | G | 144.04 | 427.50 | 19.20 | 1.51 | 22.27 | 12.70 | 4.08 | 4.41 |
| *Cleistogenes squarrosa* | IM | G | 111.86 | 463.88 | 23.20 | 1.73 | 20.39 | 14.12 | 4.51 | 5.96 |
| *Clematis tangutica* | TB | H | NA | 485.60 | 18.10 | 1.45 | 26.83 | 12.48 | 131.83 | 12.02 |
| *Convolvulus ammannii* | IM | H | NA | 424.80 | 24.90 | 1.94 | 17.06 | 12.85 | 19.84 | 6.96 |
| *Cremanthodium humile* | TB | H | 133.04 | 469.75 | 23.85 | 1.97 | 20.60 | 14.61 | 24.54 | 12.81 |
| *Cymbaria dahurica* | IM | H | NA | 419.60 | 29.40 | 2.74 | 14.27 | 10.73 | 13.32 | 4.68 |
| *Dasiphora parvifolia* | TB | S | NA | 466.20 | 13.90 | 0.99 | 33.54 | 14.04 | 13.80 | 5.99 |
| *Dracocephalum heterophyllum* | TB | H | 145.99 | 464.37 | 26.93 | 2.04 | 18.17 | 14.24 | 14.33 | 10.42 |
| *Elymus dahuricus* | TB | G | 113.00 | 455.60 | 22.55 | 1.51 | 20.23 | 14.93 | 23.38 | 8.27 |
| *Elymus thoroldiana* | TB | G | 110.51 | 480.10 | 18.10 | 0.99 | 26.52 | 18.28 | 20.42 | 20.66 |
| *Euphorbia fischeriana* | IM/TB | H | 127.36 | 459.75 | 26.98 | 1.5 | 18.47 | 18.91 | 20.05 | 11.37 |
| *Festuca ovina* | TB | G | NA | 461.05 | 27.20 | 1.13 | 18.12 | 23.78 | 27.17 | 20.78 |
| *Galium verum* | IM | H | 116.44 | 457.3 | 18.40 | 1.50 | 24.85 | 12.31 | 7.42 | 12.07 |
| *Gentiana burkillii* | TB | H | 145.99 | 480.00 | 28.60 | 2.18 | 18.08 | 14.62 | 18.62 | 9.70 |
| *Gentiana dahurica* | TB | H | NA | 471.65 | 22.30 | 1.48 | 21.16 | 15.81 | 51.90 | 10.92 |
| *Gentiana squarrosa* | TB | H | NA | 393.60 | 19.00 | 1.15 | 20.72 | 16.52 | 24.55 | 14.32 |
| *Gentiana szechenyii* | TB | H | NA | 465.80 | 25.20 | 2.18 | 18.48 | 11.56 | 25.12 | 10.42 |
| *Gentianopsis paludosa* | TB | H | NA | 497.10 | 33.50 | 1.28 | 14.84 | 26.17 | 25.7 | 9.85 |
| *Glycyrrhiza uralensis* | IM | H | 132.49 | 493.50 | 38.60 | 2.62 | 12.78 | 14.71 | 39.88 | 8.37 |
| *Gypsophila davurica* | IM | H | 145.85 | 419.30 | 28.40 | 1.45 | 14.76 | 19.60 | 2.58 | 6.42 |
| *Haloxylon ammodendron* | TB | S | 199.55 | 470.70 | 21.30 | 0.90 | 22.10 | 23.67 | 19.95 | 7.77 |
| *Haplophyllum dauricum* | IM | H | 120.93 | 463.60 | 32.30 | 1.56 | 14.35 | 20.65 | 5.14 | 10.88 |
| *Haplophyllum tragacanthoides* | IM | H | 116.99 | 453.80 | 24.70 | 1.30 | 18.37 | 19.00 | 5.59 | 10.92 |
| *Hedysarum fruticosum* | IM | H | 146.67 | 457.50 | 30.00 | 1.75 | 15.25 | 17.12 | 26.85 | 8.05 |
| *Hedysarum sikkimense* | TB | H | 166.80 | 453.20 | 17.65 | 2.35 | 25.69 | 11.15 | 21.15 | 10.17 |
| *Heteropappus bowerii* | TB | H | NA | 449.50 | 17.75 | 1.16 | 25.90 | 15.39 | 14.52 | 13.22 |
| *Hippolytia trifida* | IM | S | 115.84 | 427.90 | 37.60 | 1.64 | 11.38 | 22.95 | 5.17 | 14.60 |
| *Hippuris vulgaris* | TB | H | NA | 474.40 | 26.60 | 2.17 | 17.83 | 12.26 | 19.50 | 11.78 |
| *Inula britanica* | IM | H | NA | 450.10 | 26.40 | 1.58 | 17.05 | 16.69 | 20.48 | 15.38 |
| *Iris ensata* | IM | H | NA | 435.20 | 19.3 | 1.88 | 22.55 | 10.24 | 22.95 | 7.01 |
| *Iris goniocarpa* | TB | H | 84.41 | 469.40 | 21.30 | 1.70 | 22.04 | 12.53 | 38.90 | 13.55 |
| *Iris lactea* | TB | H | 72.85 | 455.00 | 19.30 | 0.77 | 23.58 | 25.06 | 9.33 | 16.39 |
| *Iris ventricosa* | IM | H | 62.20 | 419.70 | 13.90 | 1.15 | 30.19 | 12.12 | 4.49 | 4.60 |
| *Kobresia humilis* | TB | G | 136.66 | 464.06 | 24.27 | 1.58 | 20.32 | 15.70 | 7.32 | 8.05 |
| *Kobresia littledalei* | TB | G | NA | 453.35 | 31.65 | 1.55 | 14.33 | 20.82 | 8.74 | 8.68 |
| *Kobresia pygmaea* | TB | G | 107.77 | 447.11 | 19.50 | 1.76 | 24.62 | 12.76 | 37.06 | 11.83 |
| *Kobresia robusta* | TB | G | 112.30 | 420.00 | 20.70 | 2.19 | 20.29 | 9.45 | 34.67 | 8.43 |
| *Kobresia tibetica* | TB | G | 147.91 | 484.45 | 30.63 | 2.21 | 16.31 | 14.68 | 78.97 | 13.9 |
| *Koeleria cristata* | TB | G | 96.70 | 478.50 | 25.40 | 1.12 | 18.84 | 22.68 | 26.30 | 13.20 |
| *Lagochilus ilicifolius* | IM | H | 75.42 | 428.80 | 27.4 | 1.57 | 15.65 | 17.41 | 5.16 | 5.94 |
| *Lagotis brachystachya* | TB | H | NA | 456.93 | 21.48 | 1.39 | 21.34 | 15.56 | 57.62 | 13.88 |
| *Lancea tibetica* | TB | H | 134.21 | 460.95 | 20.00 | 0.99 | 23.49 | 20.71 | 31.01 | 11.17 |
| *Leontopodium leontopodioides* | TB | H | 106 | 448.17 | 24.17 | 1.23 | 21.04 | 19.77 | 6.51 | 6.44 |
| *Leontopodium longifolium* | TB | H | NA | 464.20 | 24.30 | 1.92 | 19.10 | 12.66 | 8.51 | 6.78 |
| *Leontopodium pusillum* | TB | H | 81.40 | 466.10 | 15.90 | 1.12 | 29.31 | 14.2 | 54.95 | 23.14 |
| *Leymus chinensis* | IM | G | 88.97 | 465.79 | 25.74 | 1.60 | 18.82 | 16.97 | 2.35 | 4.60 |
| *Leymus secalinus* | TB | G | NA | 461.20 | 30.35 | 1.73 | 15.19 | 17.86 | 22.15 | 13.6 |
| *Ligularia sibirica* | TB | H | NA | 445.90 | 17.70 | 1.15 | 25.19 | 15.39 | 32.36 | 15.38 |
| *Ligularia virgaurea* | TB | H | 145.14 | 445.86 | 24.12 | 1.54 | 19.50 | 15.81 | 55.36 | 12.89 |
| *Littledalea racemosa* | TB | G | 232.72 | 457.60 | 40.00 | 2.32 | 11.44 | 17.24 | 27.54 | 10.66 |
| *Meconopsis horridula* | TB | H | NA | 461.70 | 19.80 | 1.24 | 23.32 | 15.97 | 15.49 | 5.92 |
| *Morina kokonorica* | TB | H | 161.70 | 464.60 | 19.60 | 1.05 | 23.70 | 18.67 | 31.62 | 6.81 |
| *Oxytropis aciphylla* | TB | S | 71.49 | 453.50 | 18.10 | 0.93 | 25.06 | 19.46 | 27.54 | 8.66 |
| *Oxytropis ochrocephala* | TB | H | 140.80 | 452.81 | 26.04 | 1.84 | 18.42 | 15.22 | 19.92 | 12.01 |
| *Pedicularis kansuensis* | TB | H | 90.31 | 468.50 | 22.80 | 1.20 | 20.57 | 19.08 | 45.25 | 14.30 |
| *Pedicularis kansuensis* Maxim*.* subsp. *kansuensis f. albiflora* | TB | H | NA | 480.60 | 25.30 | 1.42 | 19.00 | 17.82 | 158.49 | 30.35 |
| *Peganum multisectum* | TB | H | 107.32 | 441.30 | 14.90 | 1.03 | 29.62 | 14.47 | 16.98 | 4.37 |
| *Lamiophlomis rotata* | TB | H | 180.36 | 481.28 | 22.15 | 2.16 | 21.89 | 10.47 | 33.14 | 11.82 |
| *Pleurospermum hedinii* | TB | H | NA | 500.10 | 18.60 | 1.70 | 26.89 | 10.94 | 30.90 | 21.57 |
| *Poa annua* | IM/TB | G | 144.26 | 439.97 | 23.63 | 1.19 | 19.22 | 20.04 | 8.99 | 10.51 |
| *Pocockia ruthenia* | IM | H | 89.99 | 447.40 | 35.80 | 1.65 | 12.50 | 21.75 | 3.46 | 6.97 |
| *Polygonum amphibium* | TB | H | 103.15 | 445.10 | 31.70 | 1.93 | 14.04 | 16.42 | 19.50 | 6.28 |
| *Polygonum divaricatum* | IM | H | 102.59 | 471.85 | 21.50 | 1.70 | 22.14 | 12.63 | 116.54 | 14.57 |
| *Polygonum macrophyllum* | TB | H | NA | 450.10 | 10.60 | 0.92 | 42.46 | 11.52 | 1.82 | 2.53 |
| *Polygonum viviparum* | TB | H | NA | 458.40 | 30.45 | 2.35 | 15.32 | 14.65 | 20.02 | 9.87 |
| *Potentilla saundersiana* Royle var. *caespitosa (Lehm.)* Wolf | TB | H | 149.49 | 433.10 | 32.30 | 3.16 | 13.41 | 10.22 | 11.48 | 16.88 |
| *Potentilla acaulis* | IM/TB | H | 117.27 | 451.45 | 29.15 | 1.61 | 15.48 | 21.75 | 16.77 | 9.87 |
| *Potentilla anserina* | IM/TB | H | 169.47 | 465.60 | 24.20 | 1.25 | 19.27 | 21.80 | 47.24 | 17.74 |
| *Potentilla bifurca* | IM/TB | H | 118.40 | 436.13 | 21.27 | 2.01 | 20.63 | 10.97 | 51.68 | 13.83 |
| *Potentilla fruticosa* | TB | S | NA | 463.67 | 35.80 | 1.76 | 13.45 | 21.51 | 12.85 | 9.00 |
| *Potentilla pamrioalaica* | TB | H | NA | 446.40 | 14.20 | 3.64 | 31.44 | 3.90 | 32.36 | 19.97 |
| *Potentilla saundersiana* | TB | H | NA | 438.00 | 21.20 | 2.62 | 20.66 | 8.09 | 13.18 | 18.32 |
| *Potentilla tanacetifolia* | IM | H | 127.99 | 441.75 | 24.50 | 1.80 | 18.16 | 14.48 | 16.50 | 7.58 |
| *Ranunculus nephelogenes* | TB | H | NA | 385.80 | 14.10 | 0.91 | 27.36 | 15.49 | 7.59 | 4.77 |
| *Ranunculus tanguticus* | TB | H | NA | 426.50 | 19.30 | 1.85 | 22.10 | 10.43 | 29.51 | 13.32 |
| *Rheum australe* | TB | H | NA | 469.80 | 26.57 | 2.16 | 18.72 | 12.81 | 15.24 | 13.12 |
| *Rhodiola dumulosa* | TB | H | 159.61 | 433.57 | 20.43 | 1.33 | 22.38 | 16.27 | 23.66 | 12.61 |
| *Sanguisorba officinalis* | IM | H | 139.19 | 431.67 | 24.73 | 2.49 | 18.08 | 10.43 | 36.01 | 14.69 |
| *Saposhnikovia divaricata* | IM | H | 119.42 | 427.30 | 25.30 | 2.5 | 16.89 | 10.14 | 11.29 | 9.85 |
| *Saussurea amara* | IM | H | 138.18 | 442.35 | 25.80 | 1.91 | 17.36 | 14.40 | 9.37 | 9.43 |
| *Saussurea depsangensis* | TB | H | NA | 428.70 | 27.30 | 1.11 | 15.70 | 24.59 | 15.85 | 8.07 |
| *Saussurea ovatifolia* | TB | H | 70.33 | 407.05 | 28.50 | 1.67 | 14.57 | 17.07 | 23.08 | 13.63 |
| *Saussurea pulchra* | TB | H | 143.03 | 441.93 | 19.10 | 1.37 | 23.34 | 14.06 | 35.02 | 12.67 |
| *Saussurea pygmaea* | TB | H | 132.76 | 474.85 | 22.70 | 1.77 | 21.39 | 12.82 | 94.20 | 10.17 |
| *Saussurea stoliczkae* | TB | H | NA | 471.40 | 24.60 | 2.97 | 19.16 | 8.28 | 17.38 | 28.74 |
| *Saussurea tibetica* | TB | H | NA | 458.00 | 22.20 | 1.02 | 20.63 | 21.76 | 15.85 | 12.28 |
| *Saxifraga stolonifera* | TB | H | NA | 412.75 | 21.25 | 1.51 | 20.36 | 14.14 | 30.12 | 14.67 |
| *Saxifraga tangutica* | TB | H | NA | 482.90 | 26.10 | 1.80 | 18.50 | 14.50 | 58.88 | 10.80 |
| *Scutellaria viscidula* | IM | H | 146.53 | 473.70 | 30.20 | 1.33 | 15.69 | 22.79 | 16.28 | 18.52 |
| *Sedum tatarinowii* | IM | H | NA | 420.10 | 31.90 | 3.39 | 13.17 | 9.42 | 3.65 | 5.43 |
| *Seriphidium mongolorum* | TB | S | NA | 471.90 | 24.60 | 1.34 | 19.18 | 18.36 | 26.92 | 12.98 |
| *Serratula centauroides* | IM | H | 83.50 | 453.79 | 22.84 | 1.48 | 21.06 | 15.57 | 13.13 | 13.88 |
| *Stipa aliena* | TB | G | 134.9 | 468.83 | 21.83 | 1.29 | 21.58 | 17.26 | 40.61 | 12.59 |
| *Stipa baicalensis* | IM | H | 73.13 | 455.18 | 14.62 | 1.35 | 31.32 | 10.91 | 14.76 | 3.89 |
| *Stipa breviflora* | IM/TB | G | 133.55 | 473.94 | 28.54 | 1.52 | 17.18 | 20.28 | 26.66 | 8.78 |
| *Stipa capillacea* | TB | G | 109.53 | 469.30 | 25.10 | 1.79 | 18.70 | 14.02 | 18.20 | 16.69 |
| *Stipa chingii* | IM | G | NA | 466.10 | 16.40 | 1.10 | 28.45 | 14.98 | 46.74 | 19.40 |
| *Stipa gareosa* | IM | G | 66.90 | 460.70 | 21.10 | 1.21 | 21.83 | 17.48 | 0.11 | 2.61 |
| *Stipa grandis* | IM | G | 81.08 | 478.08 | 21.18 | 0.97 | 22.95 | 22.18 | 1.44 | 4.05 |
| *Stipa klemenzii* | IM | G | 75.45 | 469.97 | 17.37 | 1.02 | 28.29 | 16.94 | 0.37 | 2.61 |
| *Stipa krylovii* | IM/TB | G | 86.38 | 471.07 | 24.59 | 1.21 | 19.86 | 20.89 | 3.74 | 4.43 |
| *Stipa penicillata* | TB | G | NA | 454.90 | 22.10 | 1.94 | 20.58 | 11.39 | 95.5 | 13.69 |
| *Stipa purpurea* | TB | G | 109.21 | 457.64 | 22.74 | 1.78 | 21.19 | 13.46 | 42.33 | 11.00 |
| *Stipa sibiricum* | TB | G | 93.08 | 471.20 | 28.00 | 1.34 | 17.00 | 21.01 | 1.02 | 4.54 |
| *Stipa splendens* | IM | G | 62.58 | 467.85 | 24.20 | 1.20 | 19.46 | 20.34 | 0.70 | 3.05 |
| *Taraxacum mongolicum* | TB | H | 154.21 | 462.20 | 15.60 | 0.62 | 29.63 | 25.16 | 10.72 | 4.63 |
| *Thalictrum alpinum* | TB | H | NA | 476.50 | 24.30 | 2.09 | 19.85 | 12.63 | 11.29 | 6.62 |

**Table S3.** Leaf phenolics, UV absorbing compounds (UVAC) and leaf traits, including specific leaf area (SLA), leaf N, P and N:P ratio, for 5 vegetable types, 3 functional groups at site level and 9 common genera (with more than 10 species) at species-by-site level. The values indicates means± SE, with different letters above the values indicating significant difference (*P* < 0.05) among vegetable types, functional groups and genera, tested using one-way ANOVA with a Duncan *post hoc test*.

|  | **n** | **Phenolics** | **UVAC** | **SLA** | **N** | **P** | **N:P** |
| --- | --- | --- | --- | --- | --- | --- | --- |
| ***Vegetable type*** |  |  |  |  |  |  |  |
| Alpine meadow | 28 | 36.32 ± 6.67^a^ | 11.41 ± 0.70^a^ | 123.32 ± 9.04^a^ | 23.80 ± 0.72^b^ | 1.67 ± 0.07 | 15.56 ± 0.51 |
| Alpine steppe | 19 | 26.53 ± 2.66^ab^ | 11.13 ± 0.66^a^ | 95.05 ± 10.94^ab^ | 23.28 ± 0.99^b^ | 1.50 ± 0.09 | 17.10 ± 0.64 |
| Typical steppe | 23 | 6.26 ± 1.75^c^ | 5.65 ± 0.45^c^ | 97.92 ± 3.39^ab^ | 27.95 ± 1.36^a^ | 1.72 ± 0.12 | 17.80 ± 0.96 |
| Meadow steppe | 7 | 15.49 ± 4.53^bc^ | 8.53 ± 1.37^b^ | 70.78 ± 22.80^b^ | 19.91 ± 1.20^b^ | 1.42 ± 0.12 | 14.89 ± 1.63 |
| Desert steppe | 7 | 3.26 ± 1.09^c^ | 5.04 ± 0.41^c^ | 91.53 ± 5.58^ab^ | 28.09 ± 1.41^a^ | 1.84 ± 0.30 | 17.47 ± 2.32 |
| ***Functional group*** |  |  |  |  |  |  |  |
| Herbs | 105 | 25.64 ± 2.54 | 10.96 ± 0.53 | 119.79 ± 3.88 | 23.99 ± 0.62 | 1.73 ± 0.06^a^ | 15.33 ± 0.43^b^ |
| Grass | 35 | 25.07 ± 6.08 | 9.68 ± 0.89 | 114.21 ± 6.04 | 23.54 ± 0.84 | 1.51 ± 0.06^ab^ | 16.66 ± 0.68^b^ |
| Shrub | 11 | 14.38 ± 2.57 | 10.00 ± 1.28 | 119.64 ± 21.37 | 26.35 ± 2.71 | 1.36 ± 0.11^b^ | 19.40 ± 1.02^a^ |
| ***Dominant genera*** |  |  |  |  |  |  |  |
| *Saussurea* | 12 | 33.95 ± 14.23^a^ | 12.80 ± 2.28^a^ | 128.39 ± 18.13^a^ | 23.78 ± 1.36^b^ | 1.66 ± 0.16^cd^ | 15.45 ± 1.43^ab^ |
| *Astragalus* | 11 | 32.79 ± 9.99^a^ | 11.32 ± 1.58^ab^ | 122.69 ± 8.72^a^ | 22.69 ± 2.11^b^ | 2.18 ± 0.28^ab^ | 11.82 ± 1.62^b^ |
| *Kobresia* | 27 | 32.20 ± 8.81^a^ | 10.58 ± 0.98^abc^ | 131.39 ± 9.97^a^ | 24.64 ± 1.41^b^ | 1.75 ± 0.12^bcd^ | 15.12 ± 0.89^ab^ |
| *Potentilla* | 17 | 27.80 ± 6.71^ab^ | 12.81 ± 1.77^a^ | 133.50 ± 8.76^a^ | 25.45 ± 1.81^b^ | 1.90 ± 0.20^bc^ | 15.97 ± 1.95^ab^ |
| *Oxytropis* | 10 | 20.68 ± 6.06^ab^ | 11.67 ± 1.42^ab^ | 126.94 ± 17.43^a^ | 25.25 ± 2.47^b^ | 1.75 ± 0.18^bcd^ | 15.64 ± 1.78^ab^ |
| *Stipa* | 57 | 16.68 ± 3.41^ab^ | 6.79 ± 0.75^bcd^ | 82.91 ± 5.21^b^ | 22.52 ± 0.72^b^ | 1.32 ± 0.06^d^ | 18.13 ± 0.70^a^ |
| *Leontopodium* | 10 | 11.53 ± 5.13^ab^ | 7.99 ± 2.16^bcd^ | 111.89 ± 13.81^ab^ | 24.41 ± 2.59^b^ | 1.33 ± 0.11^d^ | 18.78±1.72^a^ |
| *Allium* | 20 | 6.00 ± 1.56^b^ | 6.06 ± 0.53^d^ | 99.98 ± 6.10^ab^ | 33.89 ± 2.29^a^ | 2.39 ± 0.19^a^ | 14.84 ± 0.78^ab^ |
| *Leymus* | 18 | 4.55 ± 1.54^b^ | 5.60 ± 0.73^d^ | 88.97 ± 4.07^b^ | 26.25 ± 1.23^b^ | 1.61 ± 0.11^cd^ | 17.07 ± 0.98^a^ |

**Table S4.** The correlation coefficients of phenolics and other leaf traits including SLA, leaf C, N, leaf C:N and leaf UVAC using the multiple regression PGLS under considering the regional differences.

| Leaf traits | Estimate | Std. Error | t-value | Pr (>|t|) |
| --- | --- | --- | --- | --- |
| Log SLA | 0.7236 | 0.3835 | 1.887 | 0.0663 |
| Log C | -36.9508 | 17.1468 | -2.155 | 0.0271* |
| Log N | 36.3938 | 16.6165 | 2.190 | 0.0343* |
| Log C:N | 36.1154 | 16.6992 | 2.163 | 0.0364* |
| Log UVAC | 1.5772 | 0.5769 | 2.734 | 0.0092** |
| Log C × Region | 16.2083 | 6.6194 | 2.2449 | 0.0187* |
| Log N× Region | -16.0801 | 6.5525 | -2.454 | 0.0185* |
| Log C:N× Region | -15.8540 | 6.7215 | -2.359 | 0.0232* |
| Log UVAC× Region | -0.4808 | 0.2496 | -1.926 | 0.0610 |

Signif. codes: 0 ‘***’ 0.001 ‘**’ 0.01 ‘*’ 0.05 ‘.’ 0.1 ‘ ’ 1,

Residual standard error: 1.664 on 41 degrees of freedom

Multiple R-squared: 0.5825, Adjusted R-squared: 0.4909

F-statistic: 6.356 on 9 and 41 DF, p-value: 1.368e-05

**Figure S1** Phylogeny hypothesis for 151 Tibetan Plateau and Inner Mongolian grassland plants used in trait analyses. Species sampled in Tibetan Plateau in blue, in Inner Mongolian in green, and in both regions in red. Branch lengths were estimated with the method of Grafen (1989) under a Brownian model of trait evolution and the polytomies resolved at random. The phylogeny was constructed by the online phylomatic (<http://www.phylodiversity.net/phylomatic/phylomatic.html>) and phylogenetic relationships from the Angiosperm Phylogeny Group classification (APGII, 2003).


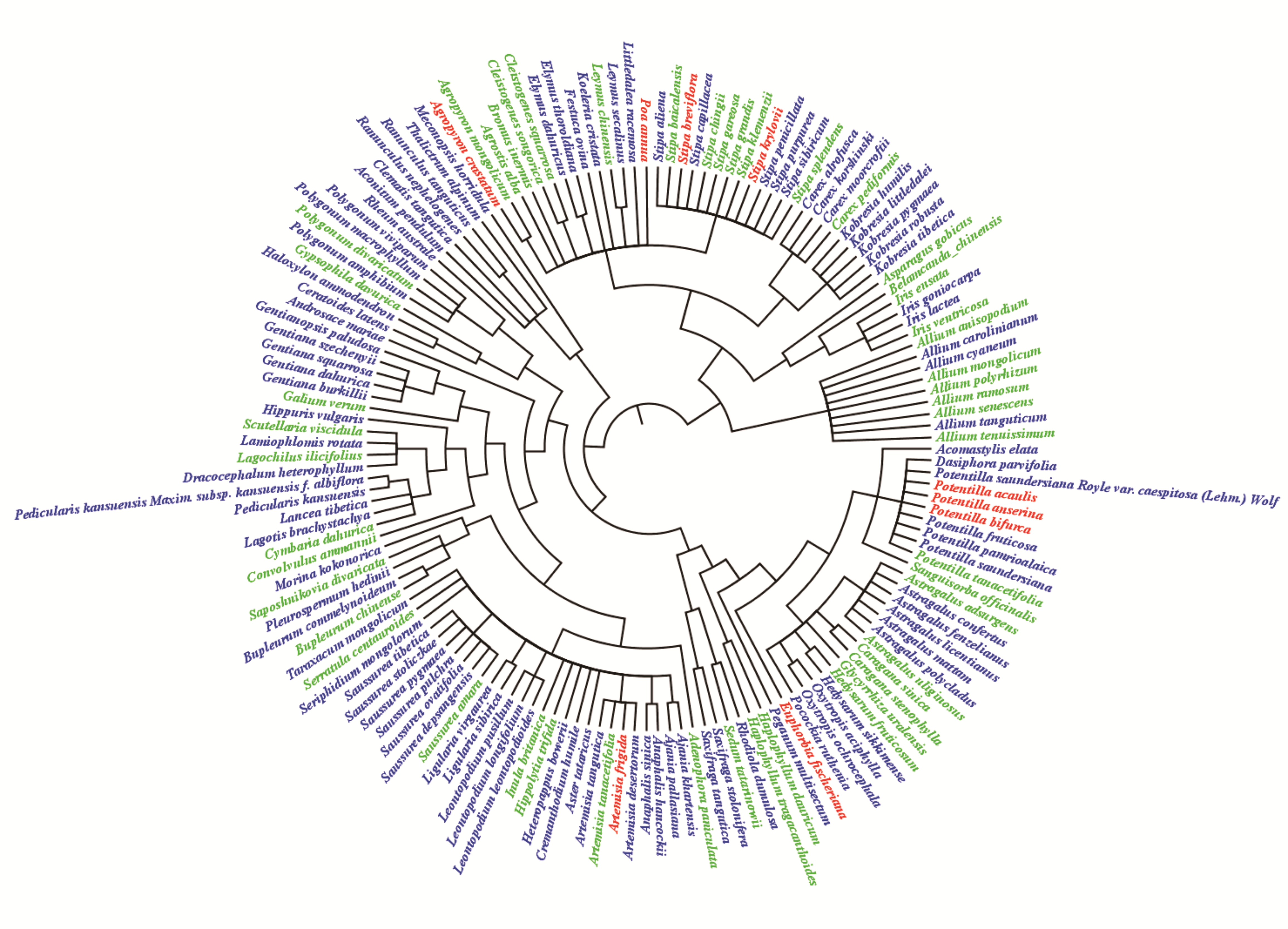

Supplement: Supplementary file 1 [file ece30003-4696-SD1.doc]
